# Suppression of Axial Tremor by Deep Brain Stimulation in Patients with Essential Tremor: Effects on Gait and Balance Measures

**DOI:** 10.5334/tohm.698

**Published:** 2022-07-01

**Authors:** Yoon Jin Choi, Basma Yacoubi, Agostina Casamento-Moran, Stefan Delmas, Bradley J. Wilkes, Christopher W. Hess, Aparna Wagle Shukla, Kelly D. Foote, David E. Vaillancourt, Michael S. Okun, Evangelos A. Christou

**Affiliations:** 1 Department of Applied Physiology and Kinesiology, University of Florida, Gainesville, FL, USA; 2 Department of Neurology, Norman Fixel Institute for Neurological Diseases, University of Florida, Gainesville, FL, USA; 3 Department of Neurosurgery, Norman Fixel Institute for Neurological Diseases, University of Florida, Gainesville, FL, USA; 4 Department of Biomedical Engineering, University of Florida, Gainesville, FL, USA; 5 Departments of Neurology and Neurosurgery, Norman Fixel Institute for Neurological Diseases, University of Florida, Gainesville, FL, USA

**Keywords:** Midline tremor, Essential tremor, Deep brain stimulation, Gait, Balance

## Abstract

**Background:**

Deep brain stimulation (DBS) of the ventralis intermedius (VIM) nucleus of the thalamus has been successful in mitigating upper limb tremor, but the effect on gait and balance performance is unclear. Here, we aim to examine the effectiveness of VIM DBS on stride length variability, sway path length, and task-relevant tremor of various body segments in essential tremor (ET).

**Methods:**

Seventeen ET individuals treated with DBS (ET DBS) and 17 age-and sex-matched healthy controls (HC) performed a postural balance and overground walking task. In separate and consecutive visits, ET DBS performed gait and balance tasks with DBS ON or OFF. The main outcome measures were sway path length, stride length variability, and tremor quantified from upper limb, lower limb, upper and lower trunk (axial) during the gait and balance tasks.

**Results:**

With DBS OFF, ET DBS exhibited significantly greater stride length variability, sway path length, and tremor during gait and balance task relative to HC. Relative to DBS OFF, DBS ON reduced stride length variability and sway path length in ET DBS. The DBS-induced reduction in stride length variability was associated with the reduction in both upper trunk tremor and upper limb tremor. The DBS-induced reduction in sway path length was associated with the reduction in upper trunk tremor.

**Discussion:**

The findings of this study revealed that VIM DBS was effective in improving gait and balance in ET DBS and that improvements in gait and postural balance were associated with a reduction of axial tremor during the tasks.

**Highlights:**

- ET patients exhibit tremor in various body locations during gait and balance.
- DBS reduced stride length variability and sway path length.
- DBS-induced improvements in gait and balance were associated with reduction in axial tremor.

## Introduction

Deep brain stimulation (DBS) of the ventralis intermedius (VIM) nucleus of the thalamus has been an effective treatment for upper limb tremor, the most noticeable symptom of essential tremor (ET) [1]. However, ET has other significant symptoms. According to a recent meta-analysis, over 40% of ET subjects exhibited gait and balance disturbances and studies have suggested that such disturbances were associated with axial tremor rather than upper limb tremor [2,3,4]. Although there is strong evidence that DBS can suppress axial tremor [5,6,7,8], its effectiveness on gait and balance performance has been debated [9,10,11]. To our knowledge, there is no study that objectively (using accelerometry) examined how VIM DBS changes tremor during gait and balance tasks (task-relevant tremor). In the current investigation, therefore, we quantified the effects of VIM DBS on tremor suppression in various body locations during gait and balance tasks and how it affected measures that associate with greater risk for falling.

Gait and balance disturbances are common in ET as evidenced from slow walking and inconsistent stride length [12,13,14]. They also exhibit worse balance scores on the Berg Balance Scale (BBS), and have less balance confidence [4,10]. Because of these gait and balance disturbances, ET subjects are at a higher risk for falls [4,15]. A hypothesis is that axial tremor would result in gait and balance disturbances in this population. This is supported by the collective data showing an association between gait and balance impairments with head tremor but not with upper limb tremor [2,3,4]. There is also further support in the finding that ET subjects with head tremor performed worse on gait and balance with a greater number of near falls [3,4]. None of these studies, however, quantified limb or axial tremor during gait or balance tasks. In addition, gait and balance disturbances can occur independent of head tremor, which suggested to our study team that tremor in other body parts (e.g. trunk) could be an important factor [16]. Therefore, it emphasizes the need of quantifying task-relevant tremor in various body segments to understand the effect of tremor on gait and balance in ET.

It is well accepted that VIM DBS is an effective therapy to suppress upper limb tremor anywhere from 50–80% [1]. However, the effectiveness of VIM DBS on gait and balance in ET remains controversial, as some studies report improvements [17,18] and other studies show no significant effect [9,11]. One possible explanation is that DBS is effective in reducing gait and balance disturbances in ET when it suppresses task-relevant axial tremor [2,3,4]. To our knowledge, only two studies have examined axial tremor and gait/balance changes with DBS [5,6]. These studies are limited because they did not quantify task-relevant tremor using accelerometry. Rather, both studies subjectively quantified axial and limb tremor using clinical scales during a postural arm task. Further, the study by Fasano et al. (2010) did not examine the association between DBS-induced axial tremor suppression and gait/balance improvements [6]. Thus, to date, it remains unknown if DBS can improve gait and balance disturbances by suppressing axial tremor.

Here, we test the hypothesis that VIM DBS will improve gait and balance performance when it suppresses axial tremor in ET. To overcome previous methodological limitations, we quantified task-relevant tremor with accelerometry and focused on stride length variability (gait) and sway path length (balance), which are strong markers for fall risk [19,20].

## Methods

### Study Population

Seventeen ET subjects undergoing VIM DBS therapy (ET DBS; 69.5 ± 8.7 years, male = 12) and 17 age- and sex- matched healthy controls (HC; 68.6 ± 7.9 years) participated in the study. The inclusion criteria for ET DBS were 1) diagnosed with ET according to the Movement Disorder Society consensus criteria [21] and had postural tremor with or without kinetic tremor; 2) undergone VIM DBS therapy with optimized DBS settings; 3) no presence of other concomitant neurological disorders. All enrolled HC reported no concomitant neurological or musculoskeletal disease. The detailed DBS settings for ET DBS are shown in Table 1. ET DBS participants performed all procedures twice: once when the DBS was ON and when the DBS was OFF. We counterbalanced the order of DBS ON/OFF testing and the two testing sessions were at least one hour apart for a washout period [6].

**Table 1:** Details of DBS setting in ET DBS subjects. Anode and cathode contacts, voltage, pulse width, and frequency for both left and right lateralized DBS for each participant.


SUBJECT	LEFT VIM OF THALAMUS				RIGHT VIM OF THALAMUS			
	
	LEAD CONTACTS	VOLTAGE (V)	PULSE WIDTH (μS)	FREQUENCY (HZ)	LEAD CONTACTS	VOLTAGE (V)	PULSE WIDTH (μS)	FREQUENCY (HZ)

1	1-2+	2.7	90	185	1-2+	2.7	90	185

2	2-3+	2.6	120	200	2-3+	3.5	120	200

3	1-C+	2.1	90	135	2-C+	2.2	90	135

4	1-C+	2.6	90	145	–	-	–	–

5	2-C+	2	90	135	2-C+	2	90	135

6	1-C+	2	90	135	1-C+	1.8	90	135

7	1+2	3.3	90	135	–	–	–	–

8	0-1-2+	2.2	180	130	–	–	–	–

9	2-C+	2.3	90	180	–	–	–	–

10	0-1+	2.7	90	135	–	–	–	–

11	1-3+	2.2	90	130	–	–	–	–

12	–	–	–	–	2-C+	2.7	104	150

13	2-3+	3.3	117	150	–	–	–	–

14	1-C+	2.2	90	145	–	–	–	–

15	2-C+	2.5	60	180	2-C+	2.6	90	130

16	2-1+	2.7	90	185	2-3+	3	90	180

17	2-3+	5	60	180	–	–	–	–


### Experimental tasks and data collection

All participants performed the following: 1) clinical assessment; 2) postural balance task; 3) overground walking.

#### Clinical assessment

Disease severity was quantified as the total scores of part A, B, and C when assessed with the Fahn-Tolosa-Marin Tremor Rating Scale (FTM-TRS) and The Essential Tremor Rating Assessment Scale (TETRAS), two commonly used clinical scales in ET [22,23]. In addition, we measured cognitive function and depression using the Montreal cognitive assessment (MoCA) and Beck depression inventory (BDI) [24,25].

#### Postural balance task

**Figure 1:**
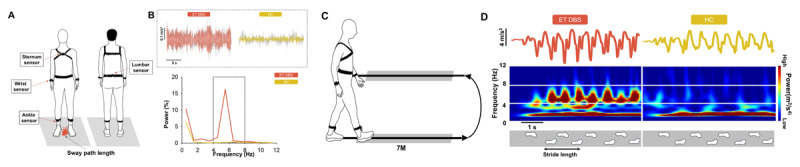
Quantification of tremor during the balance and gait tasks. **A)** Participants performed a postural balance task while standing quietly on both legs for 30 seconds and quantified their sway path length. We recorded fluctuations in acceleration from sensors placed on the sternum (upper trunk), L5 (lower trunk), left and right wrists (upper limb), and left and right ankles (lower limb). **B)** The top panel shows a representative acceleration from the sternum sensor during the middle 20 s of the postural balance task (gray) for an ET DBS subject and a HC. The red area in the acceleration signal shown in the left indicates the power from 4–8 Hz (upper trunk tremor) for an ET DBS subject when the DBS was OFF, whereas the yellow area in the right acceleration signal indicates the power from 4–8 Hz for the HC. The bottom panel shows the power spectrum of the acceleration signal for the ET DBS and HC participants. **C)** Participants walked overground for 14 m with their preferred speed. We recorded fluctuations in acceleration from the same sensors as in the postural balance task. **D)** The top panel shows the sternum acceleration during the initial 5 s of the gait task for an ET DBS subject with the DBS off (red) and a HC (yellow). The bottom panel shows the wavelet of the corresponding acceleration signal during the gait task. Tremor at each sensor location during balance and gait tasks was quantified as the power from 4–8 Hz relative to the total power from 0–12 Hz.

Participants stood quietly for 30 s with their eyes open (Figure 1A) and postural balance performance was quantified using 6 wearable sensors from APDM (Mobility Lab^TM^, APDM Inc., Portland, OR). We chose the sway path length as our main outcome variable which associates with falls [20]. Sway path length (m/s^2^) is defined as the total length of the acceleration trajectory of the lumbar sensor. Sampling rate was 128 Hz for each sensor and the data for analysis was the average of 3 trials. To determine the effect of DBS, we used the percent change of the sway path length during DBS ON compared with DBS OFF [(DBS ON – DBS OFF/DBS OFF × 100 (%)].

#### Overground walking task

Participants walked overground for 14 m at their preferred speed (Figure 1C). We focused on stride length variability, an indicator for higher risk for falls [19]. Stride length variability (%) is defined as the stride-to-stride fluctuation of the forward distance of the foot (Coefficient of variation (CV) = Standard Deviation (SD)/Mean * 100). We averaged 3 trials for the data analysis. To determine the effect of neurostimulation, we used the percent change of the stride length variability during DBS ON relative to DBS OFF.

### Tremor quantification

We quantified tremor during the postural balance task as the sum of power from 4–8 Hz in the middle 20 s of the raw acceleration signal during quiet standing for each wearable sensor (lumbar, sternum, left ankle, right ankle, left wrist, right wrist) [26,27]. To achieve this, we performed a power spectral analysis of the acceleration signal of the transverse, sagittal, and frontal planes for each body location (Figure 1B) using a customized MATLAB program (Math Works^TM^ Inc., Natick, Massachusetts, USA). For tremor during overground walking, we quantified the tremor as the sum of power from 4–8 Hz in the initial 5 s of the acceleration signal during overground walking for each wearable sensor. We performed a wavelet analysis of the raw acceleration signal for each plane (transverse, sagittal, frontal) (Figure 1D). Compared with power spectral analysis, the wavelet analysis can precisely quantify oscillations of non-stationary signals [28].

We normalized the absolute tremor (4–8 Hz) of three planes to the total power from 0–12 Hz and averaged the three planes for each trial. The mean value of three trials for each participant was used in data analysis. The upper limb tremor was quantified from the sum of both left and right wrists, the lower limb tremor from the sum of both left and right ankles, the upper trunk tremor from the sternum, and the lower trunk tremor from the lumbar sensor. To determine the effect of neurostimulation, we used the percent change of tremor during DBS ON compared with DBS OFF.

### Statistical analysis

The main dependent variables were: 1) Disease severity (FTM-TRS, TETRAS); 2) Stride length variability; 3) Sway path length; 4) Tremor during postural balance task (upper limb, lower limb, upper trunk, lower trunk); 5) Tremor during overground walking task (upper limb, lower limb, upper trunk, lower trunk).

An independent t-test compared the dependent variables and their DBS-induced changes between the two types of DBS (bilateral vs. unilateral). We then compared the dependent variables between the 17 ET DBS subjects with DBS OFF or DBS ON and their age- and sex-matched HC using an independent t-test. A paired t-test compared the dependent variables between DBS ON and DBS OFF in the 17 ET DBS subjects. The alpha level for all statistical tests was 0.05, and it was corrected for multiple comparisons using FDR correction [29].

We performed multiple regression analyses to determine which tremor locations (independent variables) associated with improvements in stride length variability and sway path length with DBS (dependent variables). In addition, we looked at the effect of DBS parameters (voltage, pulse width, frequency) on gait and balance, and tremors during the performances using regression analysis. The goodness-of-fit of the regression models was given by the squared multiple correlations (R^2^). We performed the statistical analysis using IBM SPSS Statistics 25 (IBM Corp., Armonk, NY, USA). Data are reported as means ± SD in the table and as means ± standard error in figures.

### Standard Protocol Approvals, Registrations, and Patient Consents

In accordance with the Declaration of Helsinki, all the participants signed a written informed consent prior to participating in the study. The study was approved by the Institutional Review Board of the University of Florida.

### Data Availability

Anonymized data not published within this article will be made available by request from any qualified investigator.

## Results

### Bilateral vs. Unilateral DBS

In our study, 7 out of 17 ET DBS subjects had bilateral DBS treatment. We questioned whether the improvements in gait and balance are affected by the number of implanted leads. To answer the question, we first compared disease severity, tremor, and gait and balance performances between bilateral and unilateral DBS groups. Disease severity measured with FTM-TRS and TETRAS, stride length variability, sway path length, and tremors during gait and balance were not different between the bilateral and unilateral groups with DBS OFF (all p > 0.2; see Table 2). Therefore, we combined the bilateral and unilateral groups into a single group when determining the effect of DBS on tremor suppression and gait and balance improvements.

**Table 2:** Disease severity, gait and balance performance, and tremors quantified during the gait and balance tasks comparing between unilateral and bilateral DBS treatment. There were no differences between the two DBS types. Values are shown as mean ± SD. * p < 0.05.


	UNILATERAL DBS (N = 10)		BILATERAL DBS (N = 7)	
	
	OFF	ON	OFF	ON

**Stride length variability (CV, %)**	2.6 ± 0.5	2.1 ± 0.4	2.4 ± 0.6	2.4 ± 0.6

**Sway path length (m/s** **^2^** **)**	9.8 ± 6.2	7.0 ± 3.8	9.9 ± 5.9	6.6 ± 2.1

**FTM-TRS**				

Total	47.8 ± 14.3	28.4 ± 14.9	43.4 ± 9.3	16.0 ± 7.1

Tremor	13.4 ± 5.0	9.1 ± 5.0	11.2 ± 2.9	7.8 ± 2.4

Manual	19.6 ± 7.4	12.2 ± 8.0	17.4 ± 6.8	5.5 ± 4.1

Self	14.8 ± 4.3	7.1 ± 4.6	14.7 ± 2.9	2.5 ± 2.2

**TETRAS**				

Total	49.1 ± 12.0	27.2 ± 12.4	50.0 ± 5.4	18.1 ± 7.6

Tremor	8.6 ± 3.2	6.4 ± 3.1	9.0 ± 2.8	6.1 ± 2.8

Manual	11.3 ± 3.1	7.4 ± 3.0	9.9 ± 2.7	4.4 ± 1.6

Self	29.2 ± 8.8	13.4 ± 8.5	31.0 ± 5.2	7.5 ± 4.9

**Balance tremor (%)**				

Upper limb	9.3 ± 5.8	9.1 ± 6.7	6.7 ± 5.2	7.3 ± 4.3

Lower limb	4.8 ± 3.4	4.5 ± 4.0	7.5 ± 7.8	5.8 ± 3.5

Upper trunk	7.0 ± 6.6	5.5 ± 4.9	7.0 ± 6.3	4.8 ± 1.2

Lower trunk	8.6 ± 7.6	6.3 ± 5.2	9.6 ± 7.9	6.1 ± 3.2

**Gait tremor (%)**				

Upper limb	6.5 ± 2.4	6.3 ± 3.4	7.0 ± 1.9	6.9 ± 2.1

Lower limb	8.4 ± 2.1	9.1 ± 2.5	9.1 ± 2.1	8.5 ± 2.1

Upper trunk	5.3 ± 1.3	5.0 ± 1.6	5.6 ± 1.2	5.4 ± 1.2

Lower trunk	9.0 ± 1.9	9.1 ± 2.1	9.2 ± 1.9	9.5 ± 2.6


### Effects of VIM DBS

#### DBS OFF condition

ET DBS exhibited significantly greater FTM-TRS and TETRAS scores relative to their corresponding age- and sex-matched HC (p < 0.001; Table 3). In addition, they exhibited significantly lower MoCA scores and significantly greater BDI scores than HC (p < 0.05; Table 3).

**Table 3:** Demographic characteristics, disease severity, gait and balance performance, and tremors quantified during the gait and balance tasks. Values are shown as mean ± SD. * p < 0.05. All p-values are FDR corrected.


	ET DBS OFF (N = 17)	HC (N = 17)	ET DBS ON (N = 17)	P-VALUE		

				DBS OFF VS. HC	DBS OFF VS. DBS ON	DBS ON VS. HC

**Age (yrs)**	69.5 ± 8.7	68.6 ± 7.9	–	0.4		

**Sex**	M = 12, F = 5	M = 12, F = 5	–	–		

**MoCA**	26.1 ± 1.5	27.6 ± 1.7	–	0.003*		

**BDI**	5.9 ± 6.0	2.8 ± 4.3	–	0.04*		

**Stride length variability (CV, %)**	2.6 ± 0.6	2.0 ± 0.5	2.3 ± 0.5	0.003*	0.04*	0.09

**Sway path length (m/s^2^)**	9.9 ± 6.0	4.4 ± 0.9	6.9 ± 3.2	<0.001*	0.005*	0.008*

**FTM-TRS**	46.0 ± 12.4	2.5 ± 2.4	23.3 ± 13.6	<0.001*	<0.001*	<0.001*

Part A	12.5 ± 4.3	1.8 ± 1.9	8.6 ± 4.1	<0.001*	0.001*	<0.001*

Part B	18.7 ± 7.1	0.6 ± 1.4	9.5 ± 7.4	<0.001*	<0.001*	<0.001*

Part C	14.8 ± 3.8	0.1 ± 0.3	5.2 ± 4.4	<0.001*	<0.001*	<0.001*

**TETRAS**	49.5 ± 9.6	2.4 ± 2.2	23.5 ± 11.4	<0.001*	<0.001*	<0.001*

Part A	8.8 ± 3.0	1.5 ± 1.4	6.3 ± 3.0	<0.001*	0.002*	<0.001*

Part B	10.7 ± 3.0	0.5 ± 0.9	6.2 ± 2.9	<0.001*	<0.001*	<0.001*

Part C	29.9 ± 7.4	0.4 ± 0.8	11.0 ± 7.7	<0.001*	<0.001*	<0.001*

**Balance tremor (%)**						

Upper limb	8.3 ± 5.6	7.5 ± 3.8	8.4 ± 5.8	0.5	0.43	0.49

Lower limb	6.0 ± 5.6	3.1 ± 2.1	5.1 ± 3.8	0.04*	0.38	0.09

Upper trunk	7.1 ± 6.3	4.5 ± 2.4	5.3 ± 3.8	0.3	0.31	0.49

Lower trunk	9.0 ± 7.6	4.6 ± 2.8	6.2 ± 4.4	0.05*	0.05*	0.18

**Gait tremor (%)**						

Upper limb	6.8 ± 2.2	5.0 ± 1.9	6.6 ± 2.9	0.02*	0.31	0.06

Lower limb	8.7 ± 2.1	8.2 ± 1.1	8.9 ± 2.3	0.2	0.31	0.19

Upper trunk	5.5 ± 1.3	5.2 ± 1.5	5.2 ± 1.4	0.3	0.17	0.42

Lower trunk	9.2 ± 1.9	7.9 ± 1.3	9.3 ± 2.3	0.025*	0.38	0.03*


**Figure 2:**
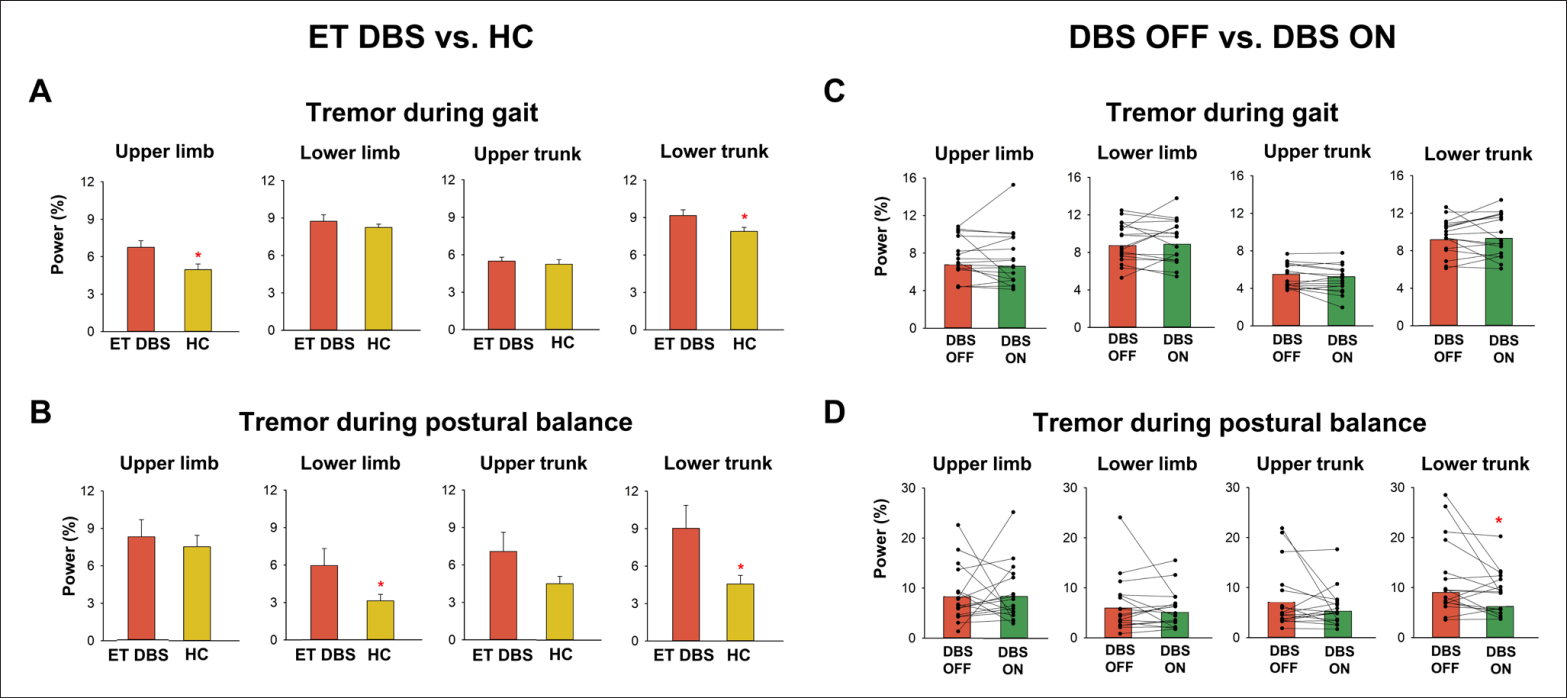
Tremor during balance and gait tasks in ET DBS subjects and HC. Relative to matched HC (yellow), ET DBS subjects with DBS OFF (red) had greater tremor in the upper limb and lower trunk during the gait task **(A)**, and had greater tremor in the lower limb and lower trunk during the postural balance task **(B)**. Relative to DBS OFF (red), DBS ON (green) did not significantly reduced tremors during the gait task **(C)**, but significantly reduced tremor in the lower trunk during postural balance task **(D)**. * p < 0.05.

Compared with HC, ET DBS exhibited greater stride length variability during the overground walking task (p = 0.003) and greater sway path length during the postural balance task (p < 0.001; Table 3). Compared with HC, ET DBS exhibited significantly greater upper limb tremor and lower trunk tremor during the walking task (p < 0.03; Figure 2A) and significantly greater lower limb tremor and lower trunk tremor during the postural balance task (p < 0.05; Figure 2B).

#### DBS ON condition

**Figure 3:**
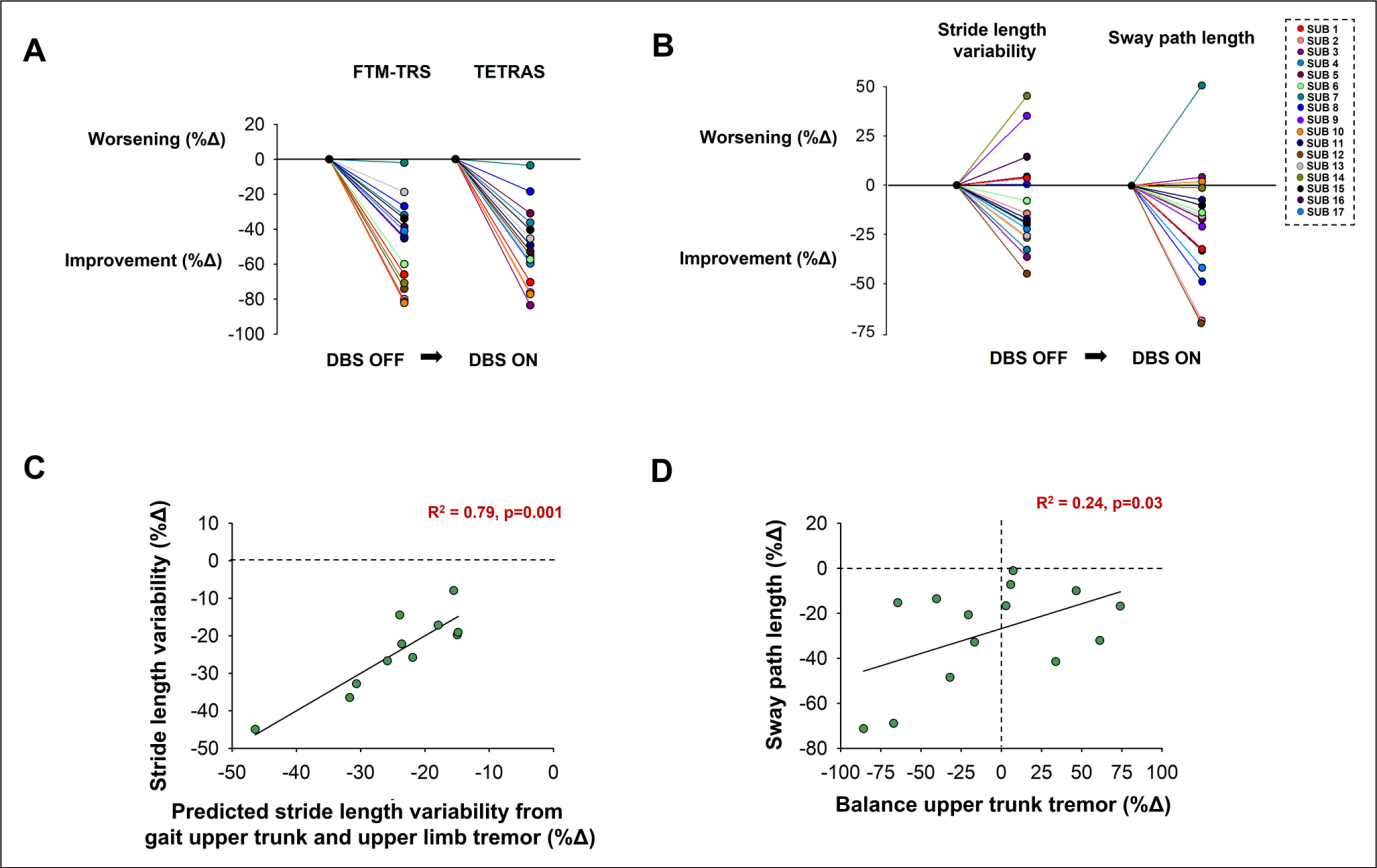
DBS-induced changes in disease severity and in gait and balance performance. **A)** DBS reduced disease severity in all 17 ET DBS subjects, as quantified with FTM-TRS and TETRAS. **B)** DBS reduced stride length variability in 11/17 ET DBS subjects and reduced sway path length in 14/17 ET DBS subjects. The relation between tremor and balance and gait performance with neurostimulation. **C)** The reduction in stride length variability with neurostimulation associated with tremor reduction in upper limb and upper trunk during the gait task. **D)** The reduction in sway path length with neurostimulation associated with tremor reduction in the upper trunk during the postural balance task. Percent change (% Δ) = ((DBS ON-OFF)/DBS OFF) × 100.

DBS ON significantly reduced FTM-TRS and TETRAS scores for all ET DBS subjects (p < 0.002, Table 3; Figure 3A). However, their FTM-TRS and TETRAS scores, remained significantly greater than HC (p < 0.001; Table 3).

DBS ON significantly reduced stride length variability during the walking task (p = 0.04; Figure 3B). The stride length variability with DBS ON was not significantly different from HC (p = 0.09; Table 3). DBS ON also reduced sway path length during the postural balance task (p = 0.005; Figure 3B). However, their sway path length remained significantly greater than HC (p = 0.008; Table 3).

Although DBS ON reduced tremor at all body locations during the walking task, these reductions were not statistically significant (Figure 2C). Similarly, DBS ON reduced tremor at all body locations during the postural balance task, but the change was statistically significant only for the lower trunk (p = 0.049; Figure 2D). Tremor at all locations and for both the walking and postural balance task were not significantly different between ET DBS and HC during the DBS ON condition (p > 0.1; Table 3).

### Association between DBS-induced changes in tremor and performance

DBS reduced stride length variability during walking in 11/17 ET DBS subjects (Figure 3B). The reduction in stride length variability was strongly associated with the reduction in tremor amplitude at the upper trunk (part r = 0.41, p = 0.01) and at the upper limb (part r = 0.3, p = 0.04) (R^2^ = 0.79, p = 0.001; Figure 3C). DBS reduced sway path length during the postural balance task in 14/17 ET DBS subjects (Figure 3B). The reduction in sway path length associated with the reduction in tremor amplitude at the upper trunk (R^2^ = 0.24, p = 0.03; Figure 3D). Results were similar when we examined individuals who had unilateral or bilateral DBS.

### Association between DBS parameters with tremor and performance

To identify the effect of DBS parameters on tremor suppression and gait and balance performance, we used a regression analysis between DBS parameters (voltage, pulse width, frequency) with disease severity, gait and balance performance, and tremors during gait and balance. We did not find any significant associations between DBS parameters with disease severity, gait and balance performance, and tremors during gait and balance both in DBS OFF and ON conditions (all R^2^ < 0.1, p > 0.1).

## Discussion

Our goal was to examine the effectiveness of VIM DBS on stride length variability, sway path length, and tremor amplitude at various body segments during gait and balance tasks. The data revealed that relative to HC, ET DBS subjects exhibited impaired gait and balance performance, as evidenced from greater stride length variability and sway path length. They also exhibited greater tremor amplitude in the upper limb and lower trunk during the walking task and greater tremor amplitude in lower limb and lower trunk during the quiet standing task. In addition, we showed that DBS improved gait and balance performance and reduced tremor primarily during the postural balance task. Most importantly, the results revealed that the largest DBS-induced decrements in stride length variability related to reductions in upper limb and upper trunk tremor, whereas the greatest DBS-induced reductions in sway path length related to reductions in upper trunk tremor. These DBS-induced improvements in gait and balance appear to be independent of lead number and stimulation characteristics. In summary, our findings suggest the possibility that trunk tremor is an important factor in the determination of gait and balance performance in ET.

### Gait and postural balance impairments in ET

A recent meta-analysis suggested that over 40% of all ET subjects exhibit gait and balance impairments [14]. Primary gait impairments include slower walking speed, longer double support time, and greater stride length variability [4,10,12]. ET subjects also exhibit impaired tandem gait, as evidenced by greater missteps and step width [10,13]. ET subjects also exhibited balance impairments, as evidenced from greater sway area, lesser limits of stability, lesser balance confidence, and reduced functional performance when assessed with BBS [3,4,9,15].

Compared with HC, ET DBS subjects exhibited greater stride length variability and sway path length. Greater stride variability may signify a reduced ability to allocate attention to control step rhythmicity [30] or a reduced coordination between the lower limbs [31]. Greater postural sway during a quiet standing task could be the result of diminished sensory ability (proprioceptive, vestibular, and visual system) [32] or increased low-frequency oscillations in the activity of the muscles for postural maintenance [33]. Regardless, both measures indicate deficits in the ability to control the body and strongly associate with a greater risk for falls in older adults [19] and patients with neurological disorders [20].

### Tremor during gait and balance tasks

We sought to understand if the presence of tremor affected stride length variability during walking and sway path length during a postural balance task. We provide novel evidence that tremor is greater in ET DBS than HC during walking and during the postural balance task at various body segments including the upper and lower limbs, as well as the upper and lower trunk. Previously, studies associated tremor during the clinical exam with gait/balance performance. For example, axial tremor was assessed by a subjective evaluation of the tongue, head, and voice tremor during rest and action, and it was later associated with gait/balance performance [3,4]. The non-significant associations reported between tremor and gait/balance performance may be explained by the tremor not being quantified during the gait or balance task.

### Neurostimulation effect

Although there is strong evidence that DBS reduces tremor [1,16], its effect on gait and balance performance has been debated. Some studies have observed no significant changes in gait and balance performance when DBS was turned ON or OFF [9,11]. In contrast, other studies have reported a reduction in the number of missteps during a tandem walk, a reduction in stride length variability during an overground walking task, and an improvement in postural sway during a postural balance task (when DBS ON) [17,18].

In this cohort of 17 ET DBS subjects, we found a significant reduction in stride length variability and postural sway. Interestingly, stride length variability reduced to the level of HC, suggesting that VIM DBS may possibly have reduced the risk of falls for these subjects although our dataset did not have a validated measure of falls to compare. Consistent with previous studies [1,16,27], DBS was effective in reducing upper limb tremor. Here, we found reduction in upper limb tremor during an overground walking and postural balance task. In addition, we showed that there was a reduction in trunk tremor, a form of axial tremor. Most importantly, we found that the DBS-induced reduction in axial tremor was associated with gait and balance performance improvements. Specifically, a reduction in upper trunk tremor was associated with a reduction in sway path length, whereas a reduction in upper trunk and upper limb tremor was associated with a reduction in stride length variability.

*Why is the reduction of trunk tremor with DBS interesting?* Trunk tremor is considered axial tremor, which is thought to be a major contributor to gait and balance disturbances in ET. Indeed, several studies show that only head tremor (axial) associates with gait and balance impairments in ET [2,3,4]. Control of the trunk will necessitate control of postural muscles (e.g. abdominal muscles or back extensors – located in the midline of the trunk) to maintain a steady whole-body posture. Tremor in the trunk, therefore, will impact the whole-body control during gait/balance tasks. Cerebellar degeneration is thought to possibly contribute to axial tremor based on indirect evidence that ET subjects with head tremor had greater cerebellar atrophy at the anterior region of vermis compared with ET subjects without head tremor [34]. Gait and balance disturbances observed in ET also have characteristics of cerebellar degeneration, but with less severity compared with cerebellar ataxia subjects [14]. Thus, it is plausible that DBS could provide walking and balance symptom relief by reducing axial tremor.

### Implications for DBS surgery

DBS and ablative therapies are among the few effective treatments for suppressing tremor in severe, drug-refractory ET cases [1]. The target area for DBS is commonly the VIM region of the thalamus. Given that the major symptom in ET is bilateral upper limb tremor, the targeted outcome following VIM DBS is usually reduction of upper limb tremor. Indeed, there is a 50–80% reduction in upper limb tremor with DBS. In contrast, the effectiveness of DBS in regulating gait and balance disturbances is variable [9,10,11,14]. One possibility is that the DBS parameters (voltage, pulse width, and frequencies) affect gait and balance. There are conflicting findings how DBS parameter modulation affects gait and balance. For example, short pulse width (< 40 µs) is associated with less stride length variability [17], whereas increased stimulation amplitude than therapeutic range increase ataxic symptoms during a tandem walk [6]. Our results show that the voltage, pulse width, and frequency did not associate with gait and balance performances. Another hypothesis is that additional lead implantation will be more effective on gait and balance. However, we found that the effectiveness of unilateral and bilateral DBS on gait, balance, and tremors during the performances was not statistically different. Our results are similar with the study by Mitchell and his colleagues (2019) which showed that most of the axial tremor (head or voice) was improved even with unilateral DBS, and bilateral DBS adds further benefit but not a significant amount as with unilateral DBS [35]. The other possibility is the placement of DBS brain leads within the thalamus, which aim to reduce upper limb tremor [16], impacted gait and balance. The location of the DBS lead may affect muscles (e.g. leg) beyond those that control the upper limb [1]. Here, we show that improvements in stride length variability and sway path length are associated with reductions in trunk tremor during walking and quiet standing. Thus, future DBS studies could explore optimal lead locations to possibly reduce both upper limb and axial tremor and hopefully to impact gait and balance.

### Limitations

Disease severity is typically moderate to severe in subjects that seek DBS therapy and thus, our findings may not apply to mild ET subjects. Although in our recent publication we show that the severity of tremor is similar between ET DBS and ET subjects with the same disease severity [27], there is evidence that neurostimulation can induce ataxic symptoms [11,36]. We cannot refute or support this assertion based on our current results because all our participants were tested post DBS surgery. In addition, the sample size for the unilateral and bilateral DBS stimulation groups was relatively small, which gives us small statistical power to address all posed questions. Thus, future studies with more subjects in each group could lead to statistically significant findings between the two groups. Nonetheless, we show that neurostimulation is beneficial for this ET DBS cohort in terms of gait and balance performances. Future studies should identify the effect of DBS surgery and compare gait and balance performance in ET with and without DBS, and with a larger sample size.

Further, we did not measure head tremor during gait and balance which is another form of axial tremor. However, we have head tremor quantified during a postural arm task from the previous study [27]. We looked at the associations between sway path length and stride length variability with head tremor, but we did not find any associations among these measures. Future studies should include head tremor during the gait and balance and see whether this is also relevant to the performance improvements.

## Conclusion

In conclusion, the findings of this study suggest that VIM DBS can improve gait and balance disturbances in ET, when it suppresses axial (trunk) tremor. Clinically, this finding is important because suppression of axial tremor can become a target for VIM DBS surgery. Future studies should identify unique lead anatomical locations in the thalamus that suppress axial tremor to improvise thalamic DBS into an effective treatment for ET patients with gait and balance problems.
